# Effect of Food and Dosing Regimen on Safety and Efficacy of Proton Pump Inhibitors Therapy—A Literature Review

**DOI:** 10.3390/ijerph18073527

**Published:** 2021-03-29

**Authors:** Agnieszka Wiesner, Małgorzata Zwolińska-Wcisło, Paweł Paśko

**Affiliations:** 1 Department of Food Chemistry and Nutrition, Faculty of Pharmacy, Jagiellonian University Medical College, 9 Medyczna Str, 30-688 Kraków, Poland; agnieszka.wiesner@doctoral.uj.edu.pl; 2 Unit of Clinical Dietetics, Department of Gastroenterology and Hepatology, Faculty of Medicine, Jagiellonian University Medical College, 2 Jakubowskiego Str, 30-688 Kraków, Poland; m.zwolinska-wcislo@uj.edu.pl

**Keywords:** proton pump inhibitors, food, meal, interactions, alcohol, juice, dosing regimen, timing, compliance, GERD

## Abstract

Proton pump inhibitors (PPIs) are the first-choice drugs used to prevent and treat acid-related diseases. However, a lack of satisfactory response to the standard PPI dose (“PPI failure”) is often reported, especially in patients with gastroesophageal reflux disease. Poor compliance seems to be one of the main causes of PPI failure; hence, it is crucial to gain knowledge on how to properly administer PPIs. In this review, we aimed to evaluate the effect of food, beverages, and dosing regimen on pharmacokinetics and pharmacodynamics of PPIs and to frame recommendations for healthcare professionals to improve both patient’s counseling and compliance to treatment with PPIs. A total of 201 papers were identified following a literature search. After full-text evaluation, 64 studies were included in the review. Co-administration of PPIs with a meal may affect both their bioavailability and effectiveness; however, the influence of food depends on the type of drug and its formulation. Except for pantoprazole, PPIs can be administered in the morning or evening; however, morning intake generally provides better daytime control of gastric acidity. In most cases, the choice of the proper schedule of administration should be based on the patient’s symptoms and individual dosing preferences.

## 1. Introduction

Proton pump inhibitors (PPIs) are the first-choice drugs prescribed (1) for the treatment of esophagitis, peptic ulcer disease (PUD), and gastroesophageal reflux disease (GERD); (2) as a part of eradication therapy for *Helicobacter pylori*; and (3) for the prevention of nonsteroidal anti-inflammatory drugs (NSAIDs)-associated ulcers. The use of PPIs without a prescription is widespread: in 2020, PPI tablets were among the five leading over-the-counter (OTC) product categories in the USA, based on sales [1].

Despite the established position of PPIs in the treatment of acid-related diseases, a lack of satisfactory response to the standard PPI dose (so-called “PPI failure”) is often reported, especially in patients with GERD [2]. It is estimated that among patients with GERD treated with PPI once daily, 10 to almost 40% of patients may still experience disease symptoms [2,3]. Failure to respond to a PPI may not only affect the patient’s quality of life but also result in increased healthcare costs (due to outpatient visits, diagnostic procedures, etc.) [4].

In a non-erosive subtype of reflux disease (NERD) PPIs can be ineffective, because symptoms are due to visceral hypersensitivity (and not the irritation by gastric acid) [5]. Poor compliance seems to be another important cause for PPI failure—it is reported that 20–50% of patients with GERD may use PPIs irregularly or administer them incorrectly [2,3,6]. For PPIs, the appropriate dosing regimen and administration with food are crucial for the optimal treatment efficacy. The PPIs are prodrugs and require activation in the secretory canaliculus of parietal cells. Because of this, the ingestion 30–60 min before a mealtime, to ensure appropriate drug concentrations ahead of proton pump activation, is needed. Consequently, the duration of therapeutic effect is partly dictated by the time of meals but also drug formulation. In a recent randomized study of 64 patients with persistent heartburn (despite treatment with 20 mg omeprazole), Waghray et al. [7] assessed whether the correction of only the dosing regimen could improve the therapeutic effectiveness. After 6 weeks of adhering to the recommended omeprazole dosing regimen, both frequency and severity scores in the GERD symptom assessment scale were significantly lower, and considerable cost savings were reported [7].

Not only patients’ but also healthcare professionals’ knowledge of mealtime-related dosing schedules of PPIs seems to be insufficient. Solem et al. [8] surveyed 501 patients and 262 physicians and revealed that although 81% of patients took their PPIs as directed, only 43% received recommendations from healthcare professionals that were compliant with the product labeling. Moreover, an alarming finding was that only 55% of patients were instructed by pharmacists on how to administer PPIs.

Our present review is aimed at evaluating the effect of food, beverages, and dosing regimen on pharmacokinetics, pharmacodynamics and clinical effectiveness of PPIs and framing recommendations for healthcare professionals to improve both patient’s counseling and compliance to treatment with PPIs.

## 2. Materials and Methods

To collect data on this topic, the authors, namely AW and PP, performed a literature search in Medline (via PubMed) and Embase databases, covering reports from 1985 to 2020. We used the following phrases and keywords during the search process: drug names (“dexlansoprazole,” “esomeprazole,” “lansoprazole,” “omeprazole,” “pantoprazole,” “rabeprazole”) in combination with “food,” “food-drug interaction,” “meal,” “breakfast,” “juice,” “alcohol,” “dosing regimen,” “timing,” “morning,” and “evening.” We also researched other resources such as Micromedex, AHFS, drugs.com, and UpToDate as well as monographs and prescribing information of particular medicinal products. Additional publications were found by checking the reference lists.

**Figure 1:**
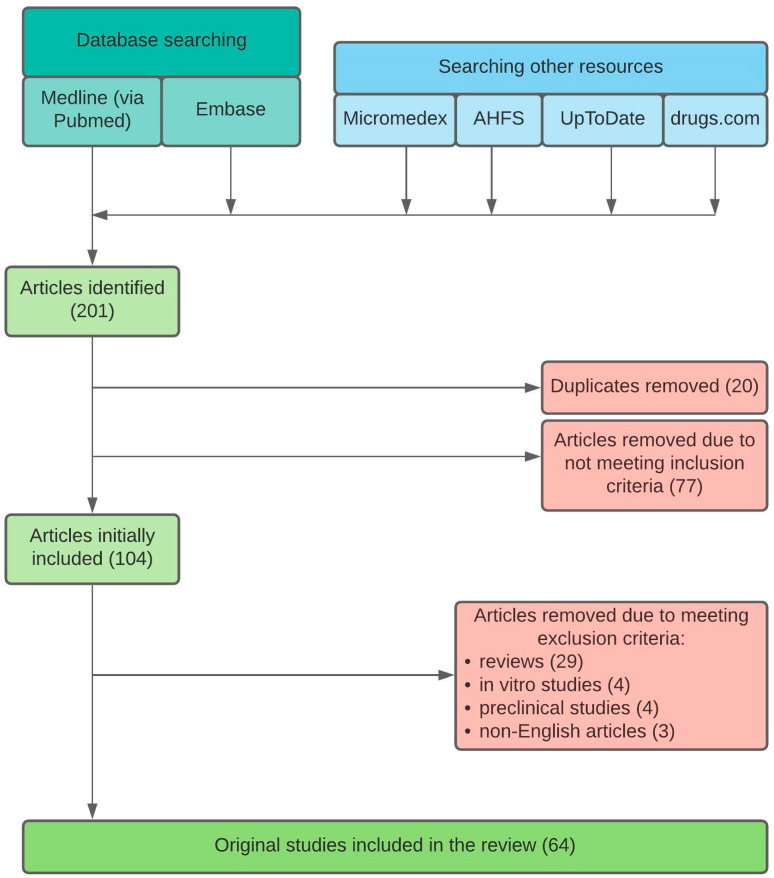
Search strategy flowchart.

All articles reporting or investigating the effect of meals, beverages, and dosing regimen on pharmacokinetics, pharmacodynamics, and clinical effectiveness of PPIs were considered for inclusion in this review. No restrictions were applied for study design, sample size, or participants’ characteristics. We initially identified a total of 201 articles. After excluding 20 duplicates and screening titles and abstracts of 181 papers, we rejected 77 articles due to not meeting inclusion criteria. Of 104 remaining articles, we excluded 29 reviews, 4 in vitro studies, 4 preclinical studies, and 3 articles written in languages other than English. Finally, 64 original clinical studies were included in this review. Figure 1 presents a flowchart of the search strategy.

## 3. Results and Discussion

### 3.1. Aspects of PPI Pharmacokinetics

To act as H^+^,K^+^-ATPase enzyme inhibitors, PPIs need to be activated in an acidic environment. Proton pump (H^+^,K^+^-ATPase) is the ultimate mediator of gastric acid secretion by parietal cells and it was proposed that activation of H^+^ secretion occurred by incorporation of H^+^,K^+^-ATPase-rich tubulovesicles into the apical plasma membrane and that the pumps were re-sequestered back into the cytoplasmic compartment on return to the resting state [9]. PPIs (omeprazole, lansoprazole, rabeprazole, pantoprazole, esomeprazole) are inactive prodrugs which are activated in the acid environment of the gastric glands. PPIs act by inhibiting this pomp, which is located on the luminal surface of gastric parietal cells. The inactive PPIs diffuse from the bloodstream into the parietal cells and subsequently into the acid environment of the secretory canaliculi, where they rearrange to form a sulfenic acid in equilibrium with a sulfenamide. Either chemical entity is then able to interact covalently with thiol groups at cysteine residues located on the luminal surface of the α-subunit of the H^+^,K^+^-ATPase. This covalent binding results in specific and essentially irreversible inactivation of the enzyme, leading to inhibition of gastric acid secretion [9,10].

However, because of acid-lability, they are prone to degradation by luminal gastric acid. Hence, PPIs are produced mainly in enteric-coated (EN) and delayed-release (DR) formulations that safely transport drugs to the place of their absorption: the proximal small bowel [11]. Drug combinations with sodium bicarbonate are also marketed to temporarily neutralize gastric pH.

Although the oral bioavailability of each PPI is different, in general, their pharmacological properties are similar [11]. All PPIs have extremely short t_½_ (approximately 1–2 h); however, the development of DR formulations solved this problem [12]. The overall binding of PPIs to plasma proteins is 95% or greater [12].

All PPIs are metabolized by intestinal and hepatic P450 cytochromes: mainly CYP2C19 and CYP3A4; hence, their interactions may theoretically occur with other CYP substrates as well as with the inhibitors and inducers of CYP isoenzymes. Nevertheless, the majority of the interactions between PPIs and drugs metabolized by CYP isoenzymes are of no clinical relevance. PPIs are known to inhibit activation of clopidogrel (prodrug metabolized by CYP2C19) and may potentially reduce its antiplatelet activity. Still, even in this case, the scientific evidence for the clinical impact of interaction is conflicting [13,14]. Pantoprazole, lansoprazole, and dexlansoprazole seem to be the least susceptible to such interactions [11].

The reason for PPI failure may be also associated with the metabolism of these drugs. The genetically determined defect in the CYP2C19 pathway was found common in certain races and interethnic variation in the capacity to metabolize PPIs should be considered during PPI pharmacotherapy. This defect may result in impaired metabolism of these drugs giving rise to three distinct phenotypes: rapid, extensive and poor metabolizers [15]. Chang et al. [16] found that AUC of omeprazole differed significantly between the three groups with a relative ratio of 1:3.7:20 between rapid, extensive and slow metabolizers. It could have a significant effect on the treatment of acid-related diseases and results in a lack of symptom relief and ineffective *H. pylori* eradication for rapid metabolizers and on the other hand over-treatment, with increased frequency of adverse effects and needless financial burden for poor metabolizers [15]. The wide interethnic variations in CYP2C19 polymorphisms were observed. Dickson and Stuart [15] reported marked interethnic variation in genotype and phenotype frequency, particularly with respect to poor metabolizers (the variation in the frequency of poor metabolizers ranged from 2.1% in Caucasians to 14.6% in Japanese) resulting in marked racial differences in the capacity to metabolize PPIs. Interethnic differences should be recognized and established by the specialist not only according to the effect and safety of therapy but also to improve cost–benefit ratio.

The presence of food generally delays the absorption of PPIs and may decrease their bioavailability [11]. However, the scope and clinical importance of this interaction strongly depend on both the type of drug and formulation; hence, we discuss the effect of food for each of the PPI representants separately.

### 3.2. Esomeprazole

Esomeprazole (syn. esomeprazole magnesium, esomeprazole strontium) is available in DR formulations: capsules, granules for oral suspension, and tablets consisting of pellets. The oral bioavailability of esomeprazole is 64% after a single 40 mg dose and 89% after repeated administration [17]. In the fasted conditions, DR tablets and capsules of esomeprazole are bioequivalent [18].

#### 3.2.1. Food Effect

Several studies have revealed that food may significantly affect the pharmacokinetic parameters of both esomeprazole magnesium and strontium formulations. Compared to fasting conditions, intake of 40 mg esomeprazole dose with food decreased AUC by 43–53% and C_max_ by 74–78%, as shown by various studies [17,18,19,20]. Additionally, Liu et al. [21] reported that concomitant ingestion of a 40 mg esomeprazole magnesium DR capsule with a meal may significantly delay drug absorption (by 2.5–3 h). The high-fat meal had 800–1000 kcal (on average 150 kcal of protein, 250 kcal of carbohydrates, and 500–600 kcal of fat).

Sostek et al. [22] performed a randomized, open-label study to compare esomeprazole pharmacokinetics when administered repeatedly under fed or fasted conditions. Forty-four healthy subjects ingested 40 mg esomeprazole capsules for 5 days, on days 1 and 5: either (1) 15 min before a high-fat meal or (2) 4 h before a standard meal, and on remaining days: 30 min before a standardized medium-fat breakfast. A high-fat meal consisted of toast with butter, hash-brown potatoes, eggs, bacon, and whole milk. On day 1, changes in esomeprazole bioavailability under fed vs. fasted conditions showed a similar pattern to that previously reported: both AUC and C_max_ decreased by 40% and 75%, respectively. However, on day 5, the effect of meal timing on both parameters was considerably lower: AUC decreased by 25% and C_max_ by 23%. Although the meal consumption shortly before esomeprazole intake significantly decreased drug bioavailability, Sostek et al. suggested that it might be clinically irrelevant during the chronic therapy. Interestingly, another study revealed that ingesting 40 mg esomeprazole dose 1 h before a high-fat meal may even increase AUC and C_max_ (by 25% and 50%, respectively) relative to the fasted conditions [20].

In two randomized, double-blind, placebo-controlled trials of 69 healthy volunteers, Furuta et al. [23,24] assessed the influence of food on esomeprazole effectiveness measured by changes in median intragastric pH and percentage time at pH > 4 (over a 24-h period and during daytime). In the first study [23], the participants were administered a single 20 mg esomeprazole capsule either 15 min before or 30 min after the supper. The supper contained 112.8 g of carbohydrates, 16.3 g of proteins, and 27.3 g of fat and had on average 762 kcal. Median intragastric pH increased slightly when esomeprazole was taken before the supper as compared to administration after a meal; however, the difference was nonsignificant. In the second study [24], the same dose and formulation of esomeprazole was ingested either (1) 30 min before or (2) 30 min after breakfast. In contrast to previous results, significant differences were found between these two regimens. When esomeprazole was taken under fed conditions, percentage time at pH > 4 was lower (over a 24-h period: 45.3% vs. 54.4% in regimen (1), during daytime: 51.4% vs. 66.5%, respectively) as well as the median intragastric pH (3.5 vs. 4.2). Furuta et al. concluded that intake of esomeprazole with food may negatively influence its inhibitory effect on gastric acid secretion. These results are in contrast to the information given in esomeprazole product monograph, where the effect of food on the acid-inhibiting activity of the drug was considered as nonsignificant [25].

In a randomized, open-label trial of 32 patients with GERD treated with 40 mg esomeprazole daily, Boltin et al. [26] evaluated whether the change in a dosing regimen with regard to food would affect treatment effectiveness. After 2 weeks of ingesting esomeprazole 30 min before breakfast, 16 of the participants were asked to switch to the administration with a standard meal for the following 4 weeks. A standard meal consisted of two slices of toast; either one egg, one piece of fruit, or 170 g yogurt; and 250 mL of tea, coffee, or juice. At the beginning of the study and after each week, patients completed standardized questionnaires: GERD frequency and severity index and the GERD-health-related quality of life (GERD-HRQL). Both groups of patients administering esomeprazole before breakfast and after a meal reported a decrease in the frequency and severity of GERD symptoms as well as improvement in the quality of life. No significant changes were observed between the study groups; hence, Boltin et al. [26] concluded that esomeprazole effectiveness is maintained after administration with a meal.

#### 3.2.2. Dosing Regimen

Wilder et al. [27] performed a randomized, cross-over study in 33 healthy volunteers to assess the effect of different esomeprazole dosing regimens on 24 h, daytime, and nighttime intragastric pH. For 5 days, the participants were given 20 mg or 40 mg esomeprazole either (1) twice daily, or once daily (2) in the morning—before breakfast, (3) in the evening—before dinner or at bedtime. Both 20 mg and 40 mg doses taken twice daily inhibited acid suppression stronger than all once-daily regimens. The 24-h intragastric pH was higher for regimen (2) than for regimen (3) (20 mg dose—4.3 vs. 3.8, 40 mg dose—4.8 vs. 4.4, respectively) as well as daytime intragastric pH (20 mg dose—4.6 vs. 3.7, 40 mg dose—4.9 vs. 4.1, respectively). Contrastingly, nighttime acid inhibition improved when esomeprazole was administered in the evening relative to the morning intake (20 mg dose—3.8 vs. 3.4, 40 mg dose—4.8 vs. 4.3, respectively). On the basis of these results, Wilder et al. suggested that the dosing regimen of esomeprazole should be chosen individually for each patient according to the symptom pattern. Maejima et al. [28] also compared the effectiveness of different esomeprazole doses and regimens of administration and concluded that esomeprazole intake twice a day may provide higher gastric acid inhibition than intake once a day in the morning.

#### 3.2.3. Administration Modes

DR esomeprazole tablets and capsules can be swallowed whole. Additionally, all the available esomeprazole formulations are allowed to be prepared and administered in a liquid form. According to the prescribing information, tablets need to be dispersed only in water [29], whereas for capsule content and granules, several other vehicles can be used [17]. Bladh et al. [30] found that the stability and dispersion features of esomeprazole granules are maintained when suspended in apple juice, orange juice, or applesauce. Additionally, in a randomized, open-label study of 41 healthy subjects, Andersson et al. [31] examined whether the ingestion of 40 mg esomeprazole magnesium capsule content with applesauce is bioequivalent to the whole capsule intake. Both administration modes resulted in comparable AUC, C_max_, t_max_, and t_½_ values. Similar results were obtained for co-intake of esomeprazole strontium capsules with applesauce [19].

### 3.3. Omeprazole

Omeprazole (syn. omeprazole magnesium) is available in immediate-release (IR) formulations: capsules and powder for oral suspension, and in DR formulations: tablets, orally disintegrating tablets (ODTs), capsules, and granules to prepare an oral suspension. The oral bioavailability of both IR and DR forms vary from 30 to 40% [32,33].

#### 3.3.1. Food Effect

According to prescribing information, the presence of food may significantly affect the bioavailability of IR omeprazole formulations. When capsules or powder for oral suspension were administered 1 h after a meal, both AUC and C_max_ decreased by 24% and 63%, respectively [32]. Liu et al. [34] performed a randomized, open-label study involving 30 healthy subjects who were given a single 40 mg IR omeprazole capsule (containing sodium bicarbonate) while fasting or with a standard meal. The meal consisted of two pieces of bread, two sausages, two eggs, 100 g of salad, and 250 mL of milk. Under fed conditions, a significant decrease in AUC_0-t_ and C_max_ (by 28% and 47%, respectively) and a delay of t_max_ (by 0.6 h) were observed relative to the fasting state. In a recent study, Ochoa et al. [35] obtained similar results; hence, both research groups suggested that IR omeprazole formulations should be administered under fasted conditions to improve the effectiveness of therapy.

The reported effect of food on the pharmacokinetics of DR omeprazole capsules is ambiguous. In a study of 17 healthy volunteers, Pillai et al. [36] revealed that after concomitant intake of light breakfast and two different brands of DR 40 mg omeprazole capsules, C_max_ decreased significantly (by 24% and 40%, respectively) and t_max_ was delayed (from 3 to 5 h for both products) However, no clinically relevant changes were detected in AUC_0–24_ and AUC_0–∞_; hence, Pillai et al. concluded that food may delay the rate (measured by C_max_) but not the extend (measured by AUC) of omeprazole absorption. Similar statements were also made in several other studies [37,38]. Contrastingly, in a randomized, open-label trial, Vaz-da-Silva et al. [39] suggested that a meal may affect both the rate and extend of omeprazole absorption. Twenty-three healthy participants were administered 20 mg DR omeprazole capsules of two different brands either under fasting conditions or concomitantly with a high-fat breakfast. The breakfast had approximately 750 kcal (of which fat constituted on average 50%) and consisted of one slice of toast with butter, one unit of cereal with whole milk, two grilled strips of bacon, two scrambled eggs, one croissant, and a noncitrus juice. After 7 days of each dosing regimen, significant changes were observed in the pharmacokinetic profiles of both DR products: not only C_max_ decreased (by 58% and 63%), but AUC_0–12_ also decreased (by 35% and 38%). Additionally, Liu et al. [34] reported a negative effect of food on the AUC of DR omeprazole capsules.

The bioavailability of DR omeprazole ODTs is also affected by food. A significant decrease in AUC_0–∞_ and C_max_ (by 19% and 56%, respectively) was observed, while t_max_ was delayed by 2 h [40].

DR tablets appear to be the most food-resistant omeprazole formulation. In a randomized, open-label study of 58 volunteers, Thomson et al. [41] assessed the influence of food on DR omeprazole tablets repeatedly administered at the dose of 20 mg in different regimens: (1) while fasting, (2) immediately before, or (3) after a standardized breakfast. Except for the delay of t_max_ (2.2 h for (1) vs. 3.5 h for (3)), no significant changes were observed in AUC and C_max_ values between all the investigated regimens. Shinkai et al. [42] reported the nonsignificant effect of food on 20 mg omeprazole pharmacokinetic parameters (namely C_max_, AUC_0-t_, t_max_, and t_½_); however, in this study, drug formulation was not mentioned.

Hatlebakk et al. [43] evaluated whether omeprazole should be taken shortly before a meal or without it. In a study of 21 healthy subjects, a 20 mg capsule of omeprazole was administered in the morning (1) 15 min before breakfast or (2) 4 h before lunch. The breakfast contained a caloric load typical for each participant and included a piece of bread or a muffin, milk or yogurt, and coffee or tea. After 8-h intragastric pH recording, a difference in the median percentage time of pH < 4 was detected for omeprazole taken before and without food (20.1% vs. 31.4%, respectively). The obtained results indicated that omeprazole needs to be taken before a meal to maintain the optimal values of gastric acidity.

#### 3.3.2. Omeprazole and Grapefruit Juice (GFJ) Consumption

Omeprazole is metabolized to 5-hydroxyomeprazole (by hydroxylation) and omeprazole sulphone (by sulfoxidation). The enzymes involved in these metabolic reactions are CYP2C19 and CYP3A4, respectively. We found one randomized study [44] that investigated the effect of GFJ on omeprazole metabolism in 13 healthy subjects. After an overnight fast, the participants were given a single 20 mg omeprazole dose either with 300 mL of GFJ or with water. Relative to the intake with water, GFJ consumption caused a significant decrease in AUC_0–12_ and C_max_ of omeprazole sulphone (by 20% and 19%, respectively). Consequently, the index of CYP3A4 activity (AUC ratio of omeprazole sulphone to omeprazole) also decreased by 33%. No significant differences were detected in the AUC of omeprazole and 5-hydroxyomeprazole as well as in the t_max_ and t_½_ of both omeprazole and its two metabolites. It was concluded that CYP3A4, but not CYP2C19, activity is inhibited by concomitant intake of omeprazole with GFJ. However, a year later, Mouly et al. [45] pointed out several limitations of the abovementioned study, indicating that even if the authors were correct, the clinical significance of GFJ-omeprazole interaction remains unclear.

#### 3.3.3. Interaction with Cranberry Juice

In vitro studies revealed that cranberry juice constituents may act as antiadhesive agents on *H. pylori*. In a randomized double-blind study, Shmuely et al. [46] confirmed that in female patients, the addition of 250 mL of cranberry juice to triple therapy with omeprazole, amoxicillin, and clarithromycin can increase the rate of *H. pylori* eradication to even 95.2%. However, Saltzman et al. [47] emphasized that ingesting cranberry juice together with omeprazole may significantly reduce gastric pH for 1 h after co-administration, and consequently, omeprazole efficacy might be altered. Hence, regular but not occasional consumption of cranberry juice should be avoided by patients chronically treated with omeprazole and probably other PPIs as well. The effect of cranberry extract supplements on gastric acidity remains unknown.

#### 3.3.4. Omeprazole and Alcohol Consumption

Brown et al. [48] assessed whether treatment with omeprazole may influence the pharmacokinetics of ethanol. Twenty-three healthy participants consumed 4.8% beer (containing ethanol in a dose of 0.6 g/kg b.w.) with a standardized meal either (1) alone—control group or (2) after at least 2 weeks of omeprazole administration. The standardized meal consisted of pasta—tagliatelle with ham and mushrooms (382 kcal, 37.9 g of carbohydrate, 19.2 g of protein, 17 g of fat) and a dessert—yogurt with strawberry compote (193 kcal, 26.3 g of carbohydrate, 6.5 g of protein, and 6.8 g of fat). In both the control and omeprazole-treated groups, the consumption of beer with food resulted in a percentage first pass ethanol metabolism of an average of 58%. No significant changes were observed in ethanol AUC and C_max_ between the two regimens. Similar results were obtained in other smaller studies, which suggests that interaction between omeprazole and ethanol is unlikely [49,50].

#### 3.3.5. Dosing Regimen

Chiverton et al. [51] performed a randomized, double-blind, placebo-controlled study to evaluate the effect of dosing regimen on omeprazole effectiveness. Six patients with duodenal ulcers were administered 20 mg omeprazole either in the morning or in the evening. After 7 days of treatment, the mean 24-h intragastric pH in the morning, evening, and control groups were as follows: 3.9 ± 1.8, 2.9 ± 1.1, and 1.7 ± 0.1, respectively. Although both omeprazole dosing schedules significantly decreased gastric acidity, omeprazole intake in the morning was found to be the most effective. In another study, Prichard et al. [52] made analogous observations in eight healthy subjects; however, the authors emphasized that during nighttime, both morning and evening omeprazole administration provided comparable control of gastric pH.

Hendel et al. [53] obtained interesting results in a study of 17 patients with GERD who were administered 40 mg omeprazole once daily (1) in the morning or (2) in the evening. After 14 days of each dosing schedule, a 24-h intragastric pH measurement was performed. After morning omeprazole administration, daytime intragastric pH was 0.72 higher than that observed after evening dosing. Contrastingly, after evening omeprazole intake, nighttime intragastric pH was 0.64 higher than that noted after morning intake. Moreover, patients’ outcomes and preferences differed depending on the symptomatology of GERD. Those with reflux induced by physical activity benefited from the morning regimen and preferred it; those with mainly nocturnal symptoms, had their reflux abolished either after only evening dose or after both morning and evening dose, however, favored evening regimen. Hendel et al. concluded that the timing of omeprazole administration may have a significant effect on both the 24-h intragastric pH profile and therapeutic effects; hence, GERD symptomatology and patient’s preferences should be considered while choosing the most appropriate dosing regimen.

It was observed that most patients who chronically administer PPIs still experience nocturnal acid breakthrough (NAB)—nighttime periods with intragastric pH < 4 lasting for 1 h or more. In order to prevent NAB, Hatlebakk et al. [54] evaluated which dosing regimen of omeprazole would most efficiently suppress the nocturnal gastric acidity. Eighteen healthy subjects were administered 40 mg omeprazole for 7 days in three dosing regimens: (1) 40 mg once daily—before breakfast, (2) 40 mg once daily—before dinner, or (3) 20 mg twice daily—before breakfast and dinner. The percentage time of nighttime intragastric pH < 4 for each dosing regimen was as follows: (1) 66.3%, (2) 31.3%, and (3) 20.5%, respectively; this indicated that both administration after dinner and splitting omeprazole dosing are more effective than morning omeprazole intake in controlling nighttime gastric acidity and consequently in preventing NAB. Additionally, Howden et al. [55] indicated that the antisecretory effect of IR formulation was faster than that observed with DR-omeprazole and a bedtime dose assures a better control of nocturnal acid secretion than lansoprazole or esomeprazole.

#### 3.3.6. Administration Modes

Both IR capsules and DR omeprazole tablets should be ingested whole [32,33]. All the remaining formulations can be prepared in a liquid form for administration. Apart from water, applesauce was tested as a vehicle for DR capsule content [33]. The results were dose-dependent: concomitant ingestion of applesauce and a DR 20 mg omeprazole capsule decreased the mean C_max_ value by 25% relative to consumption without applesauce; however, no significant change was detected for 40 mg dose. Moreover, AUC of both omeprazole doses remained unaffected; hence, co-administration of a DR omeprazole capsule with applesauce can be safely recommended.

### 3.4. Pantoprazole

Pantoprazole (syn. pantoprazole sodium) is available in DR formulations: tablets and granules to prepare an oral suspension, with the bioavailability of 77% [56].

#### 3.4.1. Food Effect

Campos et al. [57] performed a randomized, open-label study in 98 healthy volunteers to examine the effect of food on the pharmacokinetics of two pantoprazole enteric-coated (EC) formulations. Each participant ingested a single 40 mg pantoprazole tablet while fasting or with a high-fat breakfast that contained on average 800–1000 kcal. Although the measured C_max_ values were comparable under fasted and fed conditions, the presence of food slightly decreased AUC_0–∞_ in both examined formulations (by 17% and 25%, respectively) and significantly delayed t_max_ (by 4 and 5 h, respectively).

The results of the abovementioned studies and other similar studies [35,58] suggest that concomitant intake of food may slow down pantoprazole absorption; however, the clinical relevance of this effect is questionable [59]. Probably, for this reason, the recommended administration of pantoprazole tablets with food may differ depending on the country of registration. For example, according to the prescribing information of *Protonix* (registered in the USA) and *Proto-BYK* (registered in Canada), both drugs can be taken with or without food [56,59], while pantoprazole tablets available in Poland (e.g., *Controloc*, *IPP*) are recommended to be taken 1 h before a meal [60].

Regarding granules for oral suspension formulation, the pharmacokinetic study revealed that concomitant ingestion of 40 mg pantoprazole granules with a high-fat meal may significantly delay t_max_ by 2 h and considerably reduce both AUC and C_max_ (by 29% and 51%, respectively) relative to the fasted conditions [56].

#### 3.4.2. Dosing Regimen

We found only one study that investigated the influence of dosing regimen on the effectiveness of pantoprazole. In a randomized, double-blind trial [61], 12 healthy volunteers were given 40 mg pantoprazole once a day for 1 week, before the morning or evening meal. When administered in both schedules, pantoprazole effectively increased 24-h intragastric pH relative to the baseline; however, higher 24-h median pH was observed for the morning vs. evening regimen (3.3 vs. 2.7, baseline: 1.6). Interestingly, the differences in pH between the schedules were greater for daytime period than for nighttime period. Müssig et al. [61] concluded that pantoprazole should be preferably administered in the morning.

#### 3.4.3. Administration Modes

While pantoprazole tablets need to be ingested whole, granules can be opened; this provides a convenient alternative for children or patients with dysphagia [56]. In a randomized, open-label study of 25 healthy adults, Tammara et al. [62] compared two methods of intake of 40 mg pantoprazole granules: with 5 mL (one teaspoon) of applesauce or with 5 mL of apple juice. Both administration methods resulted in comparable AUC and C_max_ values and hence can be considered bioequivalent.

### 3.5. Rabeprazole

Rabeprazole (syn. rabeprazole sodium) is available in DR formulations: tablets and capsules. The oral bioavailability is approximately 52% [63].

#### 3.5.1. Food Effect

To assess the effect of a meal on the pharmacokinetic parameters of rabeprazole tablets, Shinkai et al. [42] performed a randomized, open-label study in 12 healthy volunteers. Each of the participants ingested a single 10 mg dose of rabeprazole either while fasting or after breakfast that contained on average 712 kcal. No clinically relevant differences were detected in AUC, C_max_, and t_½_ of rabeprazole between fasted and fed conditions. However, in the presence of food, rabeprazole t_max_ was significantly delayed (by 2.5 h). Similar changes were observed in several other studies [35,63,64], thus suggesting that concomitant intake of rabeprazole with a meal may slow down the rate, but not the extend of rabeprazole tablet absorption.

In two randomized, double-blind, placebo-controlled studies that included 69 healthy subjects, Furuta et al. [23,24] investigated the influence of food on rabeprazole effectiveness measured by changes in 24-h intragastric pH and percentage time at pH > 4. In the first trial [23], the participants were administered a single 10 mg rabeprazole tablet (embedded in a gelatin capsule due to the study design) either 15 min before or 30 min after the supper. The supper contained 112.8 g of carbohydrates, 16.3 g of proteins, and 27.3 g of fat and had on average 762 kcal. In the second trial [24], a rabeprazole tablet with the same dose and formulation was ingested either 30 min before or 30 min after breakfast. In both studies, no significant differences were observed in 24-h intragastric pH and percentage time at pH > 4 for rabeprazole taken under fasted and fed conditions. Furuta et al. concluded that the timing of administration with food does not influence rabeprazole tablet effectiveness.

In contrast, the effect of food on rabeprazole capsule formulation was found to be considerable. In a randomized, open-label study of 53 healthy subjects, Thyssen et al. [65] revealed that co-administration of a 10 mg DR rabeprazole capsule and a high-fat, high-calorie meal may lead to a significant decrease in both AUC_0–∞_ and C_max_ (by 27% and 55%, respectively) and a significant delay in t_max_ as well (by 2 h).

#### 3.5.2. Dosing Regimen

In a randomized, double-blind study of 20 patients with GERD, Pehlivanov et al. [66] compared the effectiveness of morning and evening rabeprazole dosing. The participants received 20 mg rabeprazole either (1) 30 min before breakfast or (2) 30 min before dinner. At the beginning and after 7 days of study, total and nocturnal esophageal acid exposure and the mean NAB duration were determined. Relative to the morning intake, the evening schedule normalized total esophageal acid exposure in a higher number of patients (71.4% vs. 42.8%). Moreover, the evening regimen provided better control of nocturnal gastroesophageal reflux. Not only the mean NAB duration was significantly lower (3.4 ± 1.5 h vs. 4.1 ± 1.8 h), but also the number of supine reflux episodes in the 24 h period (6 vs. 28). On the basis of these results, Pehlivanov et al. recommended taking rabeprazole before the evening meal as the preferred dosing regimen for GERD patients, especially for those with nocturnal symptoms.

In contrast, in a later study of 10 healthy subjects, Miki et al. [67] suggested that post-breakfast and pre-dinner repeated intake of lower (10 mg) rabeprazole dose is equally effective. For both dosing schedules, rabeprazole exhibited a comparable gastric acid inhibitory effect measured as the percentage time of pH > 4 in a 24-h period (57.7% ± 7.5 vs. 57.0% ± 7.6, respectively).

#### 3.5.3. Administration Modes

According to the prescribing information of registered rabeprazole formulations, tablets need to be swallowed whole, while capsules should be opened and their content (granules) sprinkled onto a small amount of soft food [63]. In a randomized, open-label study of 35 healthy adults, Thyssen et al. [68] compared the pharmacokinetic parameters of 10 mg rabeprazole granules when mixed with different vehicles: (1) a strawberry-flavored suspension, (2) 15 mL of applesauce, (3) 15 mL of yogurt, (4) 5 mL of infant formula, and (5) a water suspension. No significant differences were observed in pharmacokinetic parameters between the tested administration modes; hence, Thyssen et al. concluded that all the investigated vehicles are bioequivalent.

### 3.6. Lansoprazole

Lansoprazole is available in DR formulations: capsules, ODT, and granules for oral suspension. The oral bioavailability is 80–85% [11].

#### 3.6.1. Food Effect

In a randomized, open-label study in 12 healthy men, Bergstrand et al. [69] evaluated the effect of a meal on lansoprazole pharmacokinetic parameters. The participants were given a single 30 mg lansoprazole capsule under fasted conditions or with a standardized breakfast. The meal consisted of two slices of white bread with butter, two slices of cheese (40 g), 50 g of muesli, 200 g of yogurt, 300 mL of milk, and 150 mL of tea or coffee. Concomitant intake of lansoprazole and breakfast resulted in a significant decrease in AUC and C_max_ (both by approximately 50%) and a significant delay of t_max_ (from 1.8 to 3.3 h). Bergstrand et al. proposed several possible mechanisms explaining the interaction between lansoprazole and food, such as (1) binding of drug molecules to food, (2) retarded gastric emptying in the presence of food and hence delayed drug absorption, and (3) higher gastric acidity after a meal and subsequently increased breakdown of enteric coating of the capsule. Comparable results were obtained in other similar studies [70,71], which suggested that lansoprazole capsules should not be co-administered with meals.

Fujiwara et al. [72] suggested that the food intake may also affect lansoprazole bioavailability in ODT formulation. Nine healthy subjects were administered a single 30 mg OD tablet either while fasting or 15 min after breakfast. Relative to fasted conditions, the presence of meal significantly decreased both AUC_0–∞_ and C_max_ (by 32% and 50%, respectively) and significantly delayed t_max_ (from 2 to 3.3 h).

In contrast to the abovementioned studies, Moules et al. [73] concluded that inhibition of gastric acid by lansoprazole is not altered in the presence of food. In a randomized, double-blind study, 13 healthy volunteers were given either a 30 mg lansoprazole capsule or placebo (1) before breakfast or (2) after finishing breakfast. The 24-h intragastric pH was measured before treatment and after 1 week. Median acid inhibition values and the median time of pH > 3 were comparable under fasted and fed conditions (95.4% vs. 97.4% of baseline value and 20.75 h vs. 22.07 h, respectively).

In another randomized, double-blind study in 16 healthy volunteers, Brummer et al. [74] revealed that on the first day of treatment, the median 24-h intragastric pH significantly differed for lansoprazole 30 mg capsule intake while fasting and with a meal (pH = 1.4 vs. 3). However, the food effect was not maintained after 15 days (pH = 4.1 vs. 4.3). The authors suggested that the interaction between lansoprazole and food may be important only at the beginning of therapy.

Hatlebakk et al. [43] assessed whether lansoprazole should be administered shortly before a meal or without a meal. In a pharmacodynamic study of 21 healthy volunteers, lansoprazole 20 mg capsule was taken either 15 min before breakfast or 4 h before lunch. Breakfast contained the number of calories typical for each participant and included a piece of bread or a muffin, milk or yogurt, and coffee or tea. After 8-h intragastric pH recording, the median percentage time of pH < 4 differed for lansoprazole taken shortly before and without food (14.2% vs. 48.1%, respectively). Hatlebakk et al. proposed the following explanations of this result: (1) meal intake stimulates acid secretion, and low gastric pH is essential for PPI activation, (2) after meal consumption, proton pumps are activated and recruited to the surface of parietal cells, and PPIs can block only activated proton pumps. Hence, lansoprazole should be taken shortly before a meal and not without it to maintain the optimal control of gastric juice acidity during the day.

#### 3.6.2. Lansoprazole and Grapefruit Juice Consumption

Initially, in the small intestine and subsequently in the liver, lansoprazole is transformed into two major metabolites: 5-hydroxylansoprazole and lansoprazole sulfone [75]. The hydroxylation process is catalyzed mainly by CYP2C19, while the sulfoxidation process by CYP3A4 [76]. Grapefruit juice constituents have been shown to inhibit both these enzymes in the intestine; hence, the potential risk of interaction between lansoprazole and GFJ exists.

Uno et al. [76] performed a randomized study of 21 healthy subjects to assess the effect of GFJ on the pharmacokinetics of lansoprazole and its metabolites. Thirty minutes before the intake of a 60 mg lansoprazole capsule, the participants ingested either 200 mL of water or 200 mL of freshly prepared GFJ. After GFJ consumption, no significant differences were observed in the AUC of lansoprazole metabolites as well as in C_max_, t_max_, or t_½_ of lansoprazole and its metabolites. However, relative to the water group, the AUC of lansoprazole was slightly, but significantly, lowered (by 18%) in the GFJ group. Consequently, the sulfoxidation index—the AUC ratio of lansoprazole sulfone to lansoprazole—was also considerably decreased (by 42%) by GFJ ingestion. On the basis of these results, Uno et al. concluded that GFJ may affect the formation of lansoprazole sulfone by inhibiting CYP3A4. Nevertheless, the authors suggested that this effect, occurring only in the small intestine, might not influence lansoprazole pharmacokinetics due to the relatively high drug bioavailability.

In another randomized, double-blind, placebo-controlled study, Miura et al. [75] investigated the effect of GFJ on the metabolism of lansoprazole in 18 healthy volunteers of three different CYP2C19 genotype groups: heterozygous extensive metabolizers, homozygous extensive metabolizers, and poor metabolizers. Each of the participants ingested either 200 mL of water or 200 mL of fresh GFJ and subsequently took a single 60 mg dose of lansoprazole capsule. After GFJ consumption, no significant changes were detected in C_max_ or t_½_ values of both lansoprazole and lansoprazole sulfone in all three CYP2C19 genotype groups.

#### 3.6.3. Lansoprazole and Alcohol Consumption

To evaluate the risk of interaction between lansoprazole and ethanol, Battiston et al. [77] measured the drug effect on the gastric and hepatic activity of the ethanol-metabolizing enzyme—alcohol dehydrogenase. The influence of lansoprazole on ethanol metabolism was found to be neutral; hence, the authors suggested that, in contrast to imidazole derivatives (e.g., famotidine, ranitidine), therapy with lansoprazole may be continued in patients who are unable to quit or reduce ethanol consumption.

#### 3.6.4. Dosing Regimen

To compare the effect of morning and evening lansoprazole intake on 24-h intragastric acidity, Fraser et al. [78] performed a randomized, double-blind, placebo-controlled study. For 7 days, 32 healthy subjects were administered 30 mg lansoprazole dose either (1) in the morning—30 min before breakfast or (2) in the evening—at least 2 h after a meal. The morning intake resulted in a higher decrease of 24-h intragastric acidity relative to the evening regimen (36% vs. 42% of placebo value, respectively). Interestingly, significant changes were observed between morning and evening dosing only for a daytime interval. During the night, both lansoprazole dosing regimens were equally effective in controlling gastric acidity. Fraser et al. concluded that generally, morning dosing is favorable; however, an evening regimen can be beneficial for patients with mainly nocturnal symptoms. Sanders et al. [79] obtained similar results in a former 7-day study and additionally observed that morning dosing may improve 30 mg lansoprazole bioavailability relative to the evening regimen (AUC_0–24_ (ng·mL/h): 2517 ± 1737 vs. 1065 ± 684, C_max_ (ng/mL): 1054 ± 379 vs. 381 ± 249).

In contrast to the abovementioned studies, Hongo et al. [80] suggested that morning and evening lansoprazole administration is equally effective. In a small study of eight healthy volunteers, a 30 mg lansoprazole capsule was given once a day for a week, either (1) in the morning—3 h before a meal or (2) in the evening—3 h after a meal. Next, 24-h intragastric pH and percentage time of pH > 4 were measured at the beginning of the study and after 7 days. Both dosing regimens increased intragastric pH when compared with the control group (regimen (1): pH = 4.3, control: pH = 1.6; regimen (2): pH = 4.6, control: pH = 2.1). No significant differences were found in all measured values between morning and evening lansoprazole intake. In a randomized study of 10 healthy subjects, Miki et al. [67] confirmed these results for a lower—15 mg dose of lansoprazole. When taken both after breakfast and before dinner, lansoprazole exhibited a comparable gastric acid inhibitory effect measured as the percentage time of pH > 4 in a 24-h period (55.5% ± 10.8% vs. 54.6% ± 10.7%, respectively).

#### 3.6.5. Administration Modes

The standard approach to administer lansoprazole capsules is to swallow them whole. However, this might be problematic in children and patients with dysphagia. Hence, Chun et al. [81,82] investigated whether admixing lansoprazole capsule content with various vehicles would be an appropriate administration mode. In two randomized, open-label studies in 43 healthy volunteers, a single dose of 30 mg lansoprazole was given either (1) as an intact capsule or emptied into (2) a tablespoon of yogurt, (3) a tablespoon of strained pears, (4) Ensure pudding, (5) cottage cheese, (6) 180 mL of orange juice, or (7) 180 mL of tomato juice. For lansoprazole intake with cottage cheese, a considerably higher t_max_ was observed relative to ingestion of an intact capsule (2.1 and 1.5 h, respectively). Moreover, when capsule content was administered with orange juice, AUC_0–∞_ values were lower (3429 vs. 3755 ng·mL/h), and t_max_ was significantly delayed (from 1.7 to 3 h) as compared to those for the intact capsule. However, all these changes were in an acceptable equivalence range; moreover, no other statistically significant differences in pharmacokinetic parameters were observed between the regimens. Chun et al. concluded that all investigated vehicles can be used to administer lansoprazole capsules, without a negative effect on drug bioavailability.

Regarding ODT formulation, Iwasaki et al. [83] examined the influence of intake with water on lansoprazole absorption. Twelve healthy men were administered 30 mg ODT lansoprazole with or without 150 mL of water. No significant changes in AUC_0–24_ and C_max_ were detected between both administration modes.

### 3.7. Dexlansoprazole

Dexlansoprazole is available in dual delayed-release (DDR) capsules and DR orally disintegrating tablets. Each dual delayed-release capsule of dexlansoprazole contains two different sets of enteric-coated granules that disintegrate at two different pH levels—in the duodenum and the small intestine. This allows achieving two peaks of the drug concentrations [84]. The pharmacokinetic studies of dexlansoprazole modified-release formulation showed a plasma concentration–time profile, with two distinct peaks, occurring 1–2 h and 4–5 h after dosing and what should be noted 24-h intragastric pH-recordings confirm a prolonged acid inhibition [85]. Consequently, both the therapeutic level of dexlansoprazole in blood and its gastric acid inhibitory effect are maintained longer relative to other PPIs [84]. Dexlansoprazole was found to be highly effective and superior to lansoprazole in healing erosive esophagitis [86].

#### 3.7.1. Food Effect

In a randomized, open-label, placebo-controlled study, Lee et al. [87] assessed the influence of food on the pharmacokinetic and pharmacodynamic parameters of dexlansoprazole. Forty-eight healthy volunteers were given a DDR 90 mg capsule (1) while fasting, (2) 5 min before breakfast, (3) 30 min before breakfast, or (4) 30 min after breakfast. The breakfast contained a high amount of fat and consisted of two slices of toast with butter, two strips of bacon, two eggs fried in butter, 4 oz (114 g) of hash-brown potatoes, and 8 oz (237 mL) of whole milk. The presence of food—ingested either before or after dexlansoprazole administration—increased both area under the curve (AUC) (by 9–21%) and C_max_ (by 12–31%) as compared to that in fasted conditions. Additionally, for regimen (4), t_max_ was delayed significantly (by 2 h). Lee et al. [87] also measured intragastric pH before and after dexlansoprazole administration under fasted and fed conditions. Because no clinically important differences were observed, the researchers concluded that DDR dexlansoprazole capsules can be ingested regardless of food. In another study [88], the percentage time at pH > 4 over a 24-h period was evaluated for dexlansoprazole taken while fasting and after a meal; the measured values were 64% and 57%, respectively. However, because of only a slight decrease, it was concluded that dexlansoprazole may be administered without food intake.

The effect of food was also examined for orally disintegrating tablets (ODT) of dexlansoprazole. Kukulka et al. [89] performed a randomized, open-label study in 65 healthy subjects who were administered a single 30 mg ODT of dexlansoprazole either while fasting or 30 min after a high-fat breakfast. The breakfast contained 800 to 1000 kcal, 50% of which was derived from fat. The presence of food significantly delayed t_max_ (from 4 to 6 h) and decreased C_max_ by approximately 38%; however, AUC remained unaffected relative to the fasting state.

#### 3.7.2. Dosing Regimen

In a randomized, open-label study, Lee et al. [90] examined whether different dosing regimens may influence the pharmacokinetics and pharmacodynamics of dexlansoprazole. Forty-eight healthy volunteers ingested 60 mg dexlansoprazole DDR capsule 30 min before: (1) breakfast (811 kcal); (2) lunch (714 kcal); (3) dinner (658 kcal); or (4) an evening snack (344 kcal). On the 5th day of each regimen, AUC_0-t_, C_max_, and t_max_ of dexlansoprazole and the 24-h intragastric pH were measured. Apart from the delay of t_max_ (by 2–3 h) when dexlansoprazole was taken in each regimen relative to breakfast, no other clinically significant differences in pharmacokinetic parameters were detected. Similarly, 24-h intragastric pH values were comparable for all schedules of intake; hence, Lee et al. [90] concluded that dexlansoprazole DDR capsules are equally effective regardless of the time of administration.

#### 3.7.3. Administration Modes

Generally, DDR dexlansoprazole capsules should be swallowed whole; however, it is also allowed to open them and sprinkle enteric-coated granules with 15 mL (1 tablespoon) of applesauce [88]. According to product characteristics, ODT formulation should be placed on the tongue and swallowed without or with water [91]. Nevertheless, Kukulka et al. [89] revealed that ingestion of ODT dexlansoprazole with water may lower both AUC (by 15%) and C_max_ (by 26%); hence, this administration mode should not be considered as a bioequivalent administration option.

### 3.8. Limitations of Studies

We recognized several limitations of the presented studies, similar to our previous investigation in this area [92,93] listed below:

- presence of the older studies—from the 1980s and 1990s,
- unavailable data—in some studies, PPI formulation was not mentioned, as well as the quantitative and/or qualitative meal composition, not every drug formulation was tested in the presence of food,
- the attendance of healthy volunteers in many of studies—such studies cannot be fully translated to the clinical practice,
- scarce data of PPI clinical effectiveness in the presence of food—the vast majority of studies focused on changes in pharmacokinetic parameters (e.g., AUC, C_max_, t_max_) instead of more clinically relevant endpoints, such as response to treatment, symptoms alleviation or the lesion healing, limited data of PPI clinical effectiveness in different dosing regimens—only single studies evaluated the effect of PPI dosing regimen on nighttime syndromes, whereas the majority examined the changes in pharmacodynamic parameters (e.g., 24 h intragastric pH) that cannot fully reflect the clinical practice.

Overall, it is clear that there are gaps in the knowledge on interaction with medicines for gastrointestinal diseases, especially with respect to the consequences of drug–food interactions not only in influence of clinical significance but also cost and overall impact on the population which was suggested previously [94,95].

Table 1 presents the summary of the most important information about the effect of food on the pharmacokinetics and pharmacodynamics of PPIs as well as recommendations for appropriate dosing regimen and intake concerning meals. We are convinced that knowledge on this subject is vital to professionally educate patients with the aim to improve their compliance and increase the effectiveness of PPI therapy.

## 4. Summary

The studies discussed above indicate that concomitant intake of PPIs with a meal may affect both their bioavailability and effectiveness; however, the influence of food strongly depends on the type of drug and formulation. Generally, esomeprazole, lansoprazole, and omeprazole are more vulnerable to interactions with food than dexlansoprazole, pantoprazole, and rabeprazole. DDR capsules of dexlansoprazole are the most food resistant of the available PPI formulations; hence, they can be administered with or without meals and should be the first choice for patients with poor compliance. According to the results of presented studies or product characteristics, DR tablets of all PPIs can be taken irrespectively of food as well. Nevertheless, the real importance of that recommendation is questionable, unless the possible reason for higher food resistance of DR tablets formulation will be established. What should be noted, the choice of the proper schedule of PPI administration should be based on the patient’s symptoms and individual dosing preferences.

We found evidence that PPIs (lansoprazole and omeprazole) do not interact with ethanol; thus, therapy with these drugs should not be discontinued in patients who are unable to quit or reduce alcohol consumption.

We identified several studies that investigated the effect of GFJ on lansoprazole or omeprazole bioavailability; however, the clinical relevance of this interaction remains unclear. Therefore, regular but not occasional GFJ consumption during therapy with PPIs should be avoided. A similar recommendation can be made for cranberry juice intake.

Except for pantoprazole, PPIs can be administered both in the morning or in the evening; however, morning intake generally provides better daytime control of gastric acidity. In most cases, the choice of the proper schedule of administration should be based on the patient’s symptoms and individual dosing preferences.

New formulations of PPIs, or novel long-duration PPIs such as ilaprazole, AGN 201904-Z, azeloprazole or anaprazole, have been developed recently, with a better effect on nocturnal acidity and faster symptom relief. Although they have not been introduced into the therapy yet, they may represent an opportunity for more effective treatment [96].

## Figures and Tables

**Table 1:** Summary of recommendations for appropriate intake of different formulations of proton pump inhibitors with food and the time of the day.

Drug	Formulation	Food Effect	Recommended Intake Regarding Food	Recommended Dosing Regimen
Esomeprazole	DR capsules	after single dose: ↓ AUC (by 43–53%), ↓ C_max_ (74–78%) [17,19,20,21], ↑ t_max_ (by 2.4–3 h) [16] after repeated doses: ↓ AUC (by 25%), ↓ C_max_ (by 23%) [17]	- should be taken 60 min before a meal [25] - should be swallowed whole or with content mixed with 15 mL of applesauce [19,31]	- can be administered both in the morning or in the evening [27] - dosing regimen should be individually chosen based on the patient’s syndrome pattern [27]
	DR granules for oral suspension	no studies found	- should be taken 60 min before a meal [25] - granules should be mixed with 5–15 mL of water [25]	
	DR tablets	no studies found	- can be taken with or without food [29] - should be swallowed whole or dispersed in a water [29]	
Omeprazole	IR capsules	↓ AUC (by 24 and 28%, depending on the study), ↓ C_max_ (by 47 and 63%, depending on the study) [32,34,35], ↑ t_max_ (by 0.6 h) [34,35]	- should be taken 60 min before a meal [32] - should be swallowed whole with water [32]	- generally, should be taken in the morning [51,52] - evening intake can be beneficial for patients with nocturnal acid breakthrough or nocturnal reflux [54]
	IR powder for oral suspension	↓ AUC (by 24%), ↓ C_max_ (by 63%) [32]	- should be taken 60 min before a meal [32] - packet content should be emptied into 30 mL of water [32]	
	DR orally disintegrating tablets	↓ AUC (by 19%), ↓ C_max_ (by 56%), ↑ t_max_ (by 2 h) [40]	- should be taken 60 min before a meal [40] - should be placed on the tongue to disintegrate and swallowed, with or without water [40]	
	DR capsules	↓ AUC (by 35–38%) [34], ↓ C_max_ (by 24–40% and 58–63%, depending on the study) [36,38], ↑ t_max_ (by 2 h) [36]	- should be taken 60 min before a meal [33] - should be swallowed whole or with content mixed with 15 mL of applesauce [33]	
	DR granules for oral suspension	no studies found	- should be taken 60 min before a meal [33] - should be mixed with 5–15 mL of water [33]	
	DR tablets	no significant changes in AUC and C_max_, ↑ t_max_ (by 1.3 h) [41]	- can be taken with or without food [41] - should be swallowed whole with water [41]	
Pantoprazole	DR tablets	slightly ↓ AUC (by 17–25%), no significant changes in C_max_, ↑ t_max_ (by 4–5 h) [57]	- can be taken with or without food [56,59] - should be swallowed whole with water [56,59]	- preferably should be administered in the morning [61]
	DR granules for oral suspension	↓ AUC (by 29%), ↓ C_max_ (by 51%), ↑ t_max_ (by 2 h) [56]	- should be taken 30 min before a meal [56] - content should be sprinkled into 5 mL of applesauce/apple juice [56,62] - do not mix with water or other liquids or food [56]	
Rabeprazole	DR capsules	↓ AUC (by 27%), ↓ C_max_ (by 55%), ↑ t_max_ (by 2 h) [65]	- should be taken 30 min before a meal [63] - content should be sprinkled onto a small amount of applesauce/apple juice/yoghurt/fruit or vegetable-based baby food/infant formula/pediatric electrolyte solution [63,65]	- can be administered both in the morning or in the evening [67] - evening intake can be beneficial for patients with nocturnal acid breakthrough or nocturnal reflux [66]
	DR tablets	no significant changes in AUC and C_max_, ↑ t_max_ (by 2.5 h) [42] no significant changes in median intragastric pH [23,24]	- can be taken with or without food [63] - should be swallowed whole with water [63]	
Lansoprazole	DR capsules	↓ AUC and ↓ C_max_ (both by 50%), ↑ t_max_ (by 1.5 h) [69] median intragastric pH not altered [73,74]	- should be taken 30 min before a meal [69,71] - should be swallowed whole or with content mixed with 15 mL of applesauce/yoghurt/strained pears/Ensure pudding/cottage cheese/60 mL of apple juice/orange juice/tomato juice [81,82]	- can be administered both in the morning or in the evening [67,80] morning intake, before breakfast, may improve bioavailability [79]
	DR orally disintegrating tablets	↓ AUC (by 32%), ↓ C_max_ (by 50%), ↑ t_max_ (by 1.3 h) [72]	- should be taken 30 min before a meal [71,72] - should be placed on the tongue to disintegrate and swallowed, with or without water [71]	
Dexlansoprazole	DDR capsules	not significant [87]	- can be taken with or without food [87,88] - should be swallowed whole or with content sprinkled onto 15 mL of applesauce or water [88]	- can be administered irrespective of the time of the day; however, preferably at the same time every day [90]
	DR orally disintegrating tablets	no significant changes in AUC, ↓ C_max_ (by 38%), ↑ t_max_ (by 2 h) [89]	- should be taken 30 min before a meal [91] - should be placed on the tongue to disintegrate and swallowed, preferably without water [89]	

DR—delayed-release formulation; DDR—dual-delayed-release formulation; IR—immediate-release.

## Data Availability

Data availability on the request.
